# Correction: Stante et al. Four Novel *Caudoviricetes* Bacteriophages Isolated from Baltic Sea Water Infect Colonizers of *Aurelia aurita*. *Viruses* 2023, *15*, 1525

**DOI:** 10.3390/v16121880

**Published:** 2024-12-04

**Authors:** Melissa Stante, Nancy Weiland-Bräuer, Urska Repnik, Almut Werner, Marc Bramkamp, Cynthia M. Chibani, Ruth A. Schmitz

**Affiliations:** 1Institute for General Microbiology, Christian Albrechts University, Am Botanischen Garten 1-9, D-24118 Kiel, Germany; mstante@ifam.uni-kiel.de (M.S.); nweiland@ifam.uni-kiel.de (N.W.-B.); almut-werner@web.de (A.W.); bramkamp@ifam.uni-kiel.de (M.B.); cchibani@ifam.uni-kiel.de (C.M.C.); 2Central Microscopy Facility, Christian Albrechts University, Am Botanischen Garten 1-9, D-24118 Kiel, Germany; urepnik@bio.uni-kiel.de

## Figure/Table Legend

In the original publication [1], there was a mistake in the legend for Figures 1 and 2 and Table 4. The host of the phage KMM1 is a *Staphylococcus* and not a *Pseudomonas*, as stated initially. *Staphylococcus* sp. has a new NCBI accession number (PQ151711). The correct legend appears below.
**Figure 1.** Plaque and virion morphology of isolated bacteriophages KMM1-KMM4. (**A**) Plaque morphologies were detected on MB double-agar layer plates after 16 h of incubation at 30 °C. Plaques formed on a lawn of *Staphylococcus* sp., PQ151711 (KMM1), *Citrobacter freundii*, OQ398153 (KMM2), and *Citrobacter* sp., OQ398154 (KMM3, KMM4). Scale bars represent 1 mm. (**B**) Transmission electron micrographs of phage lysates KMM1–KMM4, scale bars represent 50 nm.**Figure 2.** Infection cycles of isolated phages. One-step growth curves over 120 min were performed to calculate the latent period (green arrow) and burst size (orange arrow). (**A**) KMM1-infected *Staphylococcus* sp. (PQ151711) after 27 min with the release of 55 pfu/cell, (**B**) KMM2-infected *Citrobacter freundii* (OQ398153) after 20 min with the release of 280 pfu/cell, (**C**) KMM3- and (**D**) KMM4-infected *Citrobacter* sp. (OQ398154) after 45 and 30 min, respectively, with the release of 60 and 120 pfu/cell, respectively. Values represent the mean of three biological replicates.**Table 4.** Adsorption dynamics of bacteriophages KMM1–KMM4. Adsorption rates were determined 5 min after phage addition to the primary hosts (*Staphylococcus* sp., *Citrobacter freundii*, and *Citrobacter* sp.). The number of phages adsorbed to the cells generated a decrease in phage titer. The percentage of adsorbed phages and the adsorption constant (*k*) were calculated. Values are the mean of three biological replicates with corresponding standard deviations.

## Error in Figure/Table

In the original publication, there was a mistake in the graphical abstract as published. Initially, the host of the KMM1 phage was reported as a *Pseudomonas* strain, based on a 97% sequence identity from a preliminary genetic analysis. However, after conducting more extensive tests at the DSMZ using MALDI-TOF, 16S rRNA sequencing, and microscopy, we confirmed that the correct host is a *Staphylococcus* strain, with a 99% sequence identity. The corrected graphical abstract appears below. 

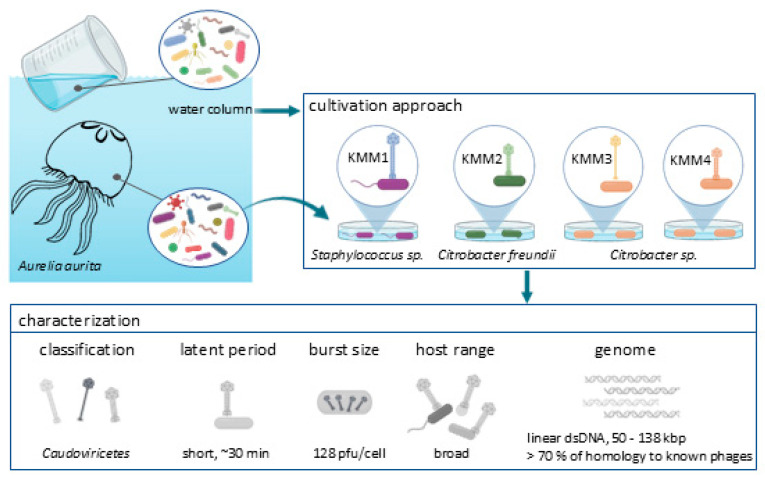



Graphical abstract

In the original publication, there was a mistake in Table 1 as published. Strains No. 8, 9, and 170, after being re-sequenced, were found to be *Staphylococcus* spp. In addition, isolate No. 8 has a new NCBI accession number (PQ151711). The corrected Table 1 appears below. 

In the original publication, there was a mistake in Table 2 as published. Initially, the host of the KMM1 phage was reported as a *Pseudomonas* strain, based on a 97% sequence identity from preliminary genetic analysis. However, after conducting more extensive tests at the DSMZ using MALDI-TOF, 16S rRNA sequencing, and microscopy, we confirmed that the correct host is a *Staphylococcus* strain, with a 99% sequence identity. We modified the name of the phage in *Staphylococcus* phage BSwM KMM1. The corrected Table 2 appears below. 

In the original publication, there was a mistake in Figure 3 as published. After re-sequence, strains 8 (NCBI Accession No. PQ151711), 9 (NCBI Accession No. OQ398155), and 170 (NCBI Accession No. OQ398167) were moved to the other Staphylococcus strains. The corrected Figure 3 appears below. 

In the original publication, there was a mistake in Figure 4 as published. The wrong KMM1 phage name was used. Phage KMM1’s name has been replaced with Staphylococcus phage BSwM KMM1. The corrected Figure 4 appears below. 

Additionally, we would like to indicate that we made the following changes in the text of the manuscript—We replaced the name of “*Pseudomonas* phage BSwM KMM1” with “*Staphylococcus* phage BSwM KMM1” in all sections of the manuscript.

In the original publication, there was an error in identifying the primary host of phage KMM1. Initially, the host of phage KMM1 was reported to be a *Pseudomonas* strain based on 97% sequence identity obtained from preliminary genetic analysis. However, after conducting more extensive testing at DSMZ using MALDI-TOF, 16S rRNA sequencing and microscopy, we confirmed that the correct host was a *Staphylococcus* strain, with a sequence identity of 99%. The primary host of the *Staphylococcus* BSwM phage KMM1 is *Staphylococcus aureus* (strain No. 8 listed in Table 1) and has the following NCBI accession number (PQ151711), which was included in all sections of the manuscript. The corrected Section 3.4 was as follows:

### 3.4. All Isolated Phages Are Highly Specific and Effective

The KMM1 phage was initially found to infect *Staphylococcus* sp., while KMM2, KMM3, and KMM4 were shown to infect *Citrobacter* spp., which are phylogenetically classified in the *Staphylococcaceae* and *Enterobacteriaceae*, respectively. The host range of the phages was determined by spot assays on 43 strains (Table 1, column “Use in the study”, category “Host range”) belonging to the same genera, *Staphylococcus* and *Citrobacter*. 

Furthermore, phages were tested against representatives of *Pseudomonadaceae*, *Enterobacteraceae* and *Rhodobacteraceae* of phylum Proteobacteria, *Streptococcaceae* of Bacilli, and *Chryseobacterium*, *Olleya*, and *Maribacter* of the abundant class of Flavobacteriia present in the *A. aurita*-associated microbiota. Bacterial sensitivity to a given bacteriophage was evaluated based on the occurrence of a lysis halo. Additionally, the respective phage efficiency of plating (EOP) was determined with those bacteria showing lysis in the spot tests. EOP for each host bacterium was calculated by comparing it with a score of 10^9^ pfu/mL obtained for the original host infection. As shown by the heatmap in Figure 3, KMM1 infects, in addition to the primary host, 15 additional strains of the Gram-positive family *Staphylococcaceae*, two of them even with a slightly higher EOP. The phages KMM2, KMM3, and KMM4 showed comparable and narrow host ranges within the genus *Citrobacter*. However, the observed phage titers and EOP were different as indicated by the color-coding dependent on the value (Figure 3). Phages KMM2 and KMM4 were further able to infect the *Enterobacteriaceae* bacterium *Shigella flexneri*. In contrast, phage KMM3 infected two *Escherichia coli* strains of *Enterobacteriaceae*. The phages infected none of the Flavobacteriia representatives.

The corrected paragraph 2–5 of Section 4 was as follows:

In this study, four phages (*Staphylococcus* phage KMM1, *Citrobacter* phages KMM2, KMM3, and KMM4) were isolated from the Baltic Sea water column (Kiel fjord) surrounding *A. aurita* individuals by a cultivation-based approach, infecting previously isolated bacteria, *Staphylococcus* and *Citrobacter*, both present in the associated microbiota of *A. aurita* [21,22]. Phages KMM1, KMM2, and KMM4 showed a clear, roundish plaque morphology, as previously described for most *Caudoviricetes* with long contractile tails (formerly known as *Myovirus*-like phages) [103]. Phage KMM3, on the other hand, showed larger plaques with a clear center surrounded by a turbid halo, commonly referred to as a “bull’s eye” plaque [104,105]. The clear halo in the plaque’s center represents the phage’s lytic activity. The turbid ring surrounding the clear halo is formed by accumulating uninfected or partially infected host bacterial cells. These cells can resist phage infection (acquired resistance, defense systems) or have only been partially infected, potentially based on the aging of the bacterial lawn (non-infective after log phase), associated increases in the size of microcolonies making up the bacterial lawn, or because of less general phenomena such as the lysis inhibition phenotype [51,106]. However, it is important to note that phage plaque morphology can vary depending on the specific phage–host system and experimental conditions [107,108].

*Citrobacter* spp., classified as Gram-negative bacteria, are widely distributed in marine environments, including seawater, sediments, and marine eukaryotes [109–112]. Although little is known about the specific ecological roles of *Citrobacter* in marine environments, they are known to contribute to the degradation of organic matter [113]. *Staphylococcus* species in marine habitats play roles in various biological processes, such as biofilm formation and interactions with other marine microbes, and can indirectly contribute to nutrient cycling [114–116]. These bacteria often serve as indicators of pollution and pose public health risks, particularly due to their potential to harbor antibiotic resistance [117–119]. Frequently introduced into marine environments by human activities, both *Staphylococcus* and *Citrobacter* can persist and spread, impacting both ecosystem and human health [120,121]. As opportunistic pathogens, they can also cause infections in marine multicellular organisms [110,111,114,122,123]. However, *Staphylococcus* and *Citrobacter* species are generally not considered critical for the health of marine ecosystems [124,125]. Their presence in marine habitats is often linked to runoff or sewage discharge from human activities, rather than any significant ecological functions within the marine environment [126,127]. Consequently, virulent phages that infect these bacteria might help balance ecosystem and metaorganism homeostasis. Bacteriophages that target *Staphylococcus* and *Citrobacter* have been identified in marine environments and may play important roles in regulating bacterial populations [2,128]. These phages can control the abundance of their host bacteria, potentially limiting the spread of pathogenic or contaminant strains like *Staphylococcus aureus* and *Citrobacter* species [2,129]. By modulating the population sizes of these bacteria, phages contribute to microbial community dynamics, influence nutrient cycling, and help maintain ecological balance in marine ecosystems [129].

Moreover, the four isolated phages reflect narrow host range phages, infecting only a limited number of bacterial strains or species [80,130]. This specificity allows them to modulate bacterial populations by selectively limiting certain strains, such as *S. aureus*, which can otherwise spread uncontrollably and pose risks to marine ecosystems [131]. This targeted regulation is crucial for maintaining ecological balance and preventing the proliferation of potential pathogens [132]. Additionally, these phages can influence horizontal gene transfer by facilitating or inhibiting the movement of genetic material between bacteria, thereby affecting the spread of antibiotic resistance genes [133]. Due to these capabilities, narrow host range phages are valuable not only in natural ecosystems but also as potential tools in phage therapy to combat resistant infections [134,135]. Furthermore, their specificity makes them ideal candidates for bioengineering applications, such as designing targeted antimicrobials or developing biosensors [132,135,136]. 

All phages identified in this study showed effective and efficient lysis of *Staphylococcus* and *Citrobacter* by fast and effective binding of the phage to the host cells, short latency periods, and high burst sizes (Tables 3 and 4). These characteristics are further important features for affecting natural microbiomes and are particularly relevant for potential therapeutic applications. Phage therapy uses intact natural phages or phage compounds to treat bacterial infections [137]. Due to the growing number of antibiotic-resistant bacterial species and the ban on the use of antibiotics in the aquatic environment [78,138–140], the interest in phage therapy particularly for aquaculture increased during the last few decades [141–143]. Phage therapy relies on extraordinary qualities of phages, including host specificity, self-replication, wide distribution, and safety [43,137,142,144]. Since phages are a natural way of managing bacterial infections, their usage does not contribute to the development of antibiotic resistance or the deposition of harmful residues in the environment. Finally, phages are versatile since they may be used alone or in cooperation with antibiotics or other therapies to improve their potency against bacterial infections. These features are entirely applicable in aquaculture, where traditional approaches to deal with pathogenic bacteria, such as antibiotics, are impossible [145,146]. Building on these advantages, the narrow host range of the isolated KMM1-KMM4 phages offers targeted therapeutic options in aquaculture, where specific bacterial infections require precise management [147,148]. These phages are effective against particular strains of *Staphylococcus* or *Citrobacter*, allowing for focused intervention without affecting non-target bacteria [129]. Their ability to selectively infect and reduce populations of specific pathogens minimizes the risk of disrupting beneficial microbial communities [136,149]. However, for these phages to be practical in aquaculture, their effectiveness must be verified against further various bacterial strains, and their stability, safety, and cost-effective production under various environmental conditions, such as pH and temperature, need to be thoroughly evaluated.

## References

Some parts of the text have been added, moved, and/or revised, and we included appropriate citations, with new numbered references [114–121,123–125,128,129,131–136,147–150]. With this correction, the order of some references has been adjusted accordingly.
114.Shineh, G.; Mobaraki, M.; Perves Bappy, M.J.; Mills, D.K. Biofilm formation, and related impacts on healthcare, food processing and packaging, industrial manufacturing, marine industries, and sanitation—A review. *Appl. Microbiol.*
**2023**, *3*, 629–665.115.Zammuto, V.; Rizzo, M.G.; Spano, A.; Spagnuolo, D.; Di Martino, A.; Morabito, M.; Manghisi, A.; Genovese, G.; Guglielmino, S.; Calabrese, G. Effects of crude polysaccharides from marine macroalgae on the adhesion and biofilm formation of Pseudomonas aeruginosa and Staphylococcus aureus. *Algal Res.*
**2022**, *63*, 102646.116.Sentenac, H.; Loyau, A.; Leflaive, J.; Schmeller, D.S. The significance of biofilms to human, animal, plant and ecosystem health. *Funct. Ecol.*
**2022**, *36*, 294–313.117.Singh, A.K.; Kaur, R.; Verma, S.; Singh, S. Antimicrobials and antibiotic resistance genes in water bodies: Pollution, risk, and control. *Front. Environ. Sci.*
**2022**, *10*, 830861.118.Jampani, M.; Mateo-Sagasta, J.; Chandrasekar, A.; Fatta-Kassinos, D.; Graham, D.W.; Gothwal, R.; Moodley, A.; Chadag, V.M.; Wiberg, D.; Langan, S. Fate and transport modelling for evaluating antibiotic resistance in aquatic environments: Current knowledge and research priorities. *J. Hazard. Mater.*
**2023**, *461*, 132527.119.Lajqi Berisha, N.; Poceva Panovska, A.; Hajrulai-Musliu, Z. Antibiotic Resistance and Aquatic Systems: Importance in Public Health. *Water*
**2024**, *16*, 2362.120.Sarkar, S.; Kamle, M.; Bharti, A.; Kumar, P. Antibiotic-resistant bacteria risks and challenges for human health and environment: An overview. *World J. Environ. Biosci.*
**2023**, *12*, 26–34.121.Ferheen, I.; Spurio, R.; Marcheggiani, S. Vehicle transmission of antibiotic-resistant pathogens mediated by plastic debris in aquatic ecosystems. *iScience* **2024**, *27*, 110026.123.Schilcher, K.; Horswill, A.R. Staphylococcal biofilm development: Structure, regulation, and treatment strategies. *Microbiol. Mol. Biol. Rev.*
**2020**, *84*, 10–1128.124.Nogales, B.; Lanfranconi, M.P.; Piña-Villalonga, J.M.; Bosch, R. Anthropogenic perturbations in marine microbial communities. *FEMS Microbiol. Rev.*
**2011**, *35*, 275–298.125.Gambino, D.; Vicari, D.; Vitale, M.; Schirò, G.; Mira, F.; Giglia, M.L.; Riccardi, A.; Gentile, A.; Giardina, S.; Carrozzo, A. Study on bacteria isolates and antimicrobial resistance in wildlife in Sicily, southern Italy. *Microorganisms*
**2021**, *9*, 203.128.Royam, M.M.; Nachimuthu, R. Isolation, characterization, and efficacy of bacteriophages isolated against Citrobacter spp. an in vivo approach in a zebrafish model (Danio rerio). *Res. Microbiol.*
**2020**, *171*, 341–350.129.Castledine, M.; Buckling, A. Critically evaluating the relative importance of phage in shaping microbial community composition. *Trends Microbiol.*
**2024**, *32*, 957–969.131.Santos, J.D.; Vitorino, I.; Reyes, F.; Vicente, F.; Lage, O.M. From ocean to medicine: Pharmaceutical applications of metabolites from marine bacteria. *Antibiotics*
**2020**, *9*, 455.132.García, P.; Madera, C.; Martinez, B.; Rodríguez, A.; Suárez, J.E. Prevalence of bacteriophages infecting Staphylococcus aureus in dairy samples and their potential as biocontrol agents. *J. Dairy Sci.*
**2009**, *92*, 3019–3026.133.Sala-Comorera, L.; Nolan, T.M.; Reynolds, L.J.; Venkatesh, A.; Cheung, L.; Martin, N.A.; Stephens, J.H.; Gitto, A.; O’Hare, G.M.; O’Sullivan, J.J. Bacterial and bacteriophage antibiotic resistance in marine bathing waters in relation to rivers and urban streams. *Front. Microbiol.*
**2021**, *12*, 718234.134.Blanco-Picazo, P.; Roscales, G.; Toribio-Avedillo, D.; Gómez-Gómez, C.; Avila, C.; Ballesté, E.; Muniesa, M.; Rodríguez-Rubio, L. Antibiotic resistance genes in phage particles from Antarctic and Mediterranean seawater ecosystems. *Microorganisms*
**2020**, *8*, 1293.135.Liu, K.; Wang, C.; Zhou, X.; Guo, X.; Yang, Y.; Liu, W.; Zhao, R.; Song, H. Bacteriophage therapy for drug-resistant Staphylococcus aureus infections. *Front. Cell. Infect. Microbiol.*
**2024**, *14*, 1336821.136.Petrovic Fabijan, A.; Lin, R.C.; Ho, J.; Maddocks, S.; Ben Zakour, N.L.; Iredell, J.R. Safety of bacteriophage therapy in severe Staphylococcus aureus infection. *Nat. Microbiol.*
**2020**, *5*, 465–472.147.Rai, S.; Kaur, B.; Singh, P.; Singh, A.; Benjakul, S.; Vijay Kumar Reddy, S.; Nagar, V.; Tyagi, A. Perspectives on phage therapy for health management in aquaculture. *Aquac. Int.*
**2024**, *32*, 1349–1393.148.Lomelí-Ortega, C.O.; Balcázar, J.L.; Quiroz-Guzmán, E. Phage therapy and aquaculture: Progress and challenges. *Int. Microbiol.*
**2023**, *26*, 439–441.149.Strathdee, S.A.; Hatfull, G.F.; Mutalik, V.K.; Schooley, R.T. Phage therapy: From biological mechanisms to future directions. *Cell*
**2023**, *186*, 17–31.150.Schackart, K.E., III; Graham, J.B.; Ponsero, A.J.; Hurwitz, B.L. Evaluation of computational phage detection tools for metagenomic datasets. *Front. Microbiol.*
**2023**, *14*, 1078760.

In addition, there are some words and content modifications throughout the text. The content of the Supplementary Materials has also been changed accordingly. The authors state that the scientific conclusions are unaffected. This correction was approved by the Academic Editor. The original publication has also been updated.

## Figures and Tables

**Figure 3 viruses-16-01880-f003:**
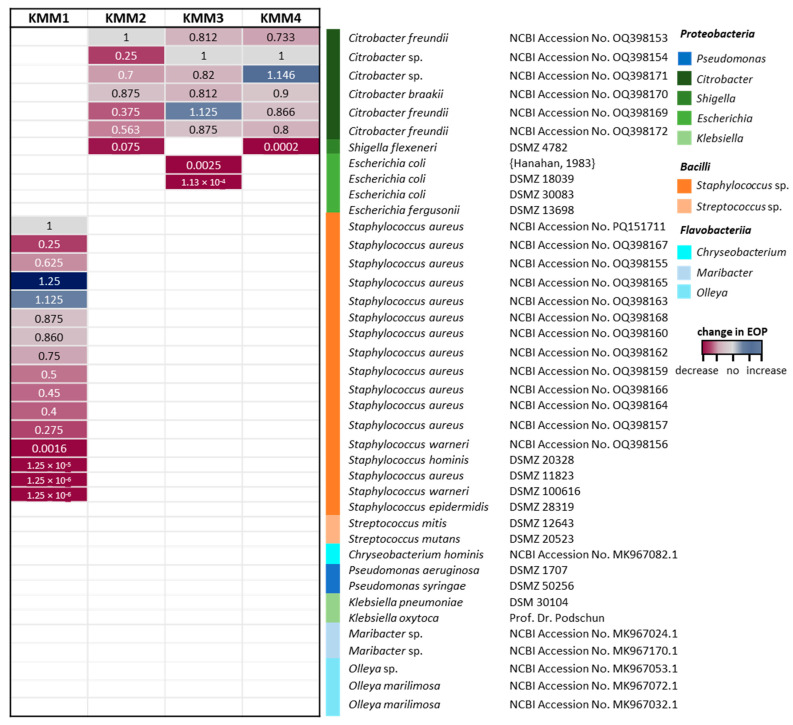
Host range of isolated phages. Phages KMM1–KMM4 were used for infection assays with selected taxons (color code on the right categorizes taxons into classes). The efficiency of plating (EOP) for each host bacterium was calculated by comparing it with a score of 10^9^ pfu/mL for the original host infection (value = 1). Missing coloring indicates no infection.

**Figure 4 viruses-16-01880-f004:**
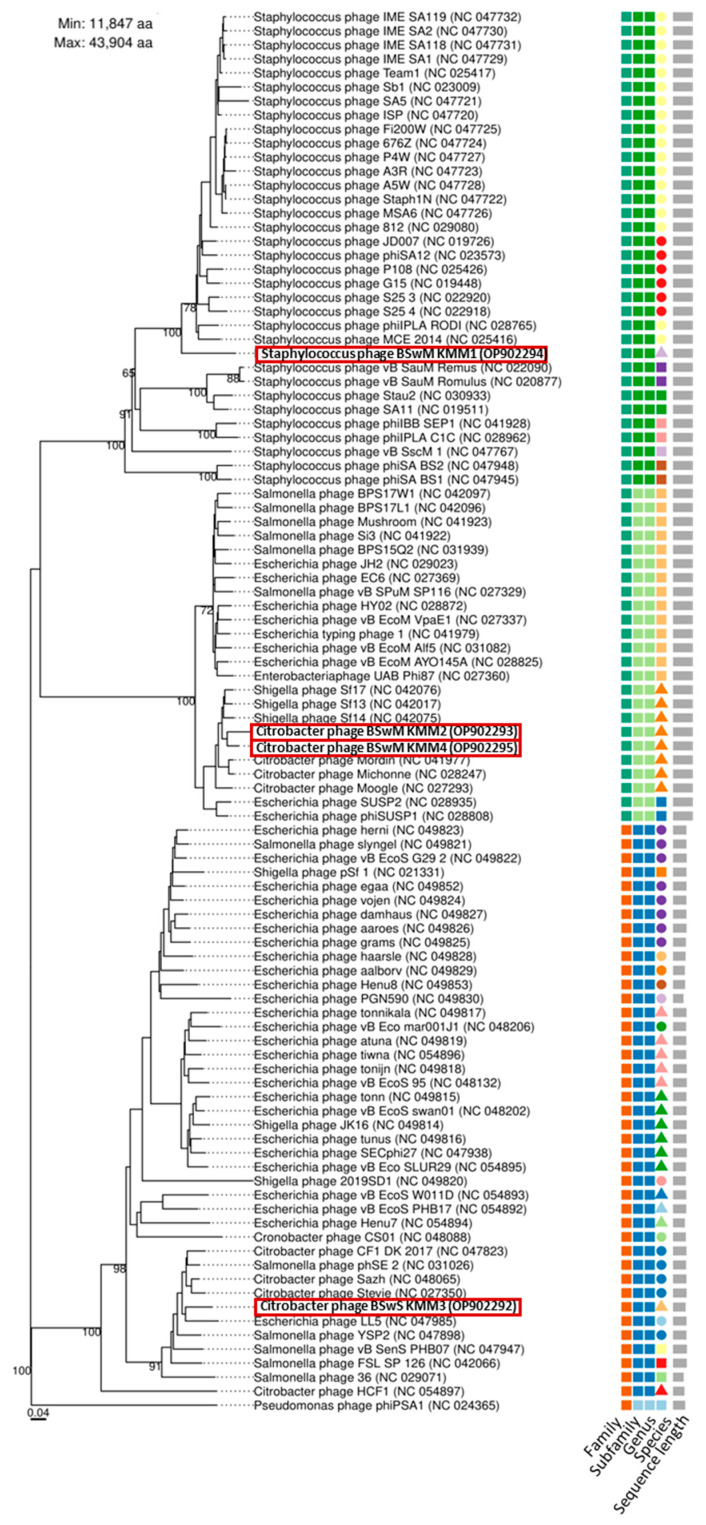
Taxonomic classification of phages KMM1–KMM4. Phylogenetic tree of isolated phages KMM1–KMM4 (red rectangles) was generated with the whole genome-based VICTOR analysis. Phages belonging to different families, subfamilies, genera, and species were color coded. The scale represents homology in %.

**Table 1 viruses-16-01880-t001:** Bacterial strains used in this study. Bacterial strains were isolated in the study [22]. Isolates are sorted by the last column and phylum level. The column “Use in This study” refers to the use of the strains in the study. If not stated differently, the listed numbers in column “Reference” reflect NCBI Accession Numbers.

Strain No.	Strain	Reference	Phylum	Class	Order	Family	Source	Growth Medium	Growth Temp.	Use in This Study
74	*Micrococcus luteus*	MK967048.1	Actinomycetota	Actinomycetia	Micrococcales	Micrococcaceae	*A. aurita* polyp Baltic Sea husbandry	Marine Bouillon	30 °C	enrichment/ first screening
75	*Arthrobacter* sp.	MK967049.1	Actinomycetota	Actinomycetia	Micrococcales	Micrococcaceae	*A. aurita* polyp Baltic Sea husbandry	Marine Bouillon	30 °C	enrichment/ first screening
83	*Gordonia terrae*	MK967057.1	Actinomycetota	Actinomycetia	Mycobacteriales	Gordoniaceae	*A. aurita* polyp Baltic Sea husbandry	Marine Bouillon	30 °C	enrichment/ first screening
15	*Sulfitobacter* sp.	MK967015.1	Pseudomonadota	Alphaproteobacteria	Rhodobacterales	Rhodobacteraceae	*A. aurita* medusa Baltic Sea	Marine Bouillon	30 °C	enrichment/ first screening
20	*Sulfitobacter pontiacus*	MK967020.1	Pseudomonadota	Alphaproteobacteria	Rhodobacterales	Rhodobacteraceae	*A. aurita* medusa Baltic Sea husbandry	Marine Bouillon	30 °C	enrichment/ first screening
23	*Sulfitobacter* sp.	MK967023.1	Pseudomonadota	Alphaproteobacteria	Rhodobacterales	Rhodobacteraceae	*A. aurita* medusa Baltic Sea husbandry	Marine Bouillon	30 °C	enrichment/ first screening
69	*Rhodobacter* sp.	MK967043.1	Pseudomonadota	Alphaproteobacteria	Rhodobacterales	Rhodobacteraceae	*M. leidyi* Baltic Sea husbandry	Marine Bouillon	30 °C	enrichment/ first screening
78	*Sulfitobacter* sp.	MK967052.1	Pseudomonadota	Alphaproteobacteria	Rhodobacterales	Rhodobacteraceae	*A. aurita* polyp Baltic Sea husbandry	Marine Bouillon	30 °C	enrichment/ first screening
86	*Ruegeria* sp.	MK967060.1	Pseudomonadota	Alphaproteobacteria	Rhodobacterales	Rhodobacteraceae	*A. aurita* polyp Baltic Sea husbandry	Marine Bouillon	30 °C	enrichment/ first screening
89	*Ruegeria* sp.	MK967063.1	Pseudomonadota	Alphaproteobacteria	Rhodobacterales	Rhodobacteraceae	*A. aurita* polyp Baltic Sea husbandry	Marine Bouillon	30 °C	enrichment/ first screening
100	*Sulfitobacter* sp.	MK967074.1	Pseudomonadota	Alphaproteobacteria	Rhodobacterales	Rhodobacteraceae	*A. aurita* polyp North Sea husbandry	Marine Bouillon	30 °C	enrichment/ first screening
117	*Ruegeria mobilis*	MK967091.1	Pseudomonadota	Alphaproteobacteria	Rhodobacterales	Rhodobacteraceae	*A. aurita* polyp North Atlantic husbandry	Marine Bouillon	30 °C	enrichment/ first screening
147	*Phaeobacter gallaeciensis*	MK967120.1	Pseudomonadota	Alphaproteobacteria	Rhodobacterales	Rhodobacteraceae	Artificial Seawater 18 PSU	Marine Bouillon	30 °C	enrichment/ first screening
188	*Sulfitobacter pseudonitzschiae*	MK967160.1	Pseudomonadota	Alphaproteobacteria	Rhodobacterales	Rhodobacteraceae	Artificial Seawater 30 PSU	Marine Bouillon	30 °C	enrichment/ first screening
13	*Bacillus cereus*	MK967013.1	Bacillota	Bacilli	Bacillales	Bacillaceae	*A. aurita* medusa Baltic Sea	Marine Bouillon	30 °C	enrichment/ first screening
16	*Bacillus* sp.	MK967016.1	Bacillota	Bacilli	Bacillales	Bacillaceae	*A. aurita* medusa Baltic Sea	Marine Bouillon	30 °C	enrichment/ first screening
17	*Bacillus cereus*	MK967017.1	Bacillota	Bacilli	Bacillales	Bacillaceae	*A. aurita* medusa Baltic Sea	Marine Bouillon	30 °C	enrichment/ first screening
19	*Bacillus* sp.	MK967019.1	Bacillota	Bacilli	Bacillales	Bacillaceae	*A. aurita* medusa Baltic Sea husbandry	Marine Bouillon	30 °C	enrichment/ first screening
76	*Bacillus weihenstephanensis*	MK967050.1	Bacillota	Bacilli	Bacillales	Bacillaceae	*A. aurita* polyp Baltic Sea husbandry	Marine Bouillon	30 °C	enrichment/ first screening
85	*Staphylococcus warneri*	MK967059.1	Bacillota	Bacilli	Bacillales	Staphylococcaceae	*A. aurita* polyp Baltic Sea husbandry	Marine Bouillon	30 °C	enrichment/ first screening
88	*Staphylococcus* sp.	MK967062.1	Bacillota	Bacilli	Bacillales	Staphylococcaceae	*A. aurita* polyp Baltic Sea husbandry	Marine Bouillon	30 °C	enrichment/ first screening
73	*Enterococcus casseliflavus*	MK967047.1	Bacillota	Bacilli	Lactobacillales	Enterococcaceae	*A. aurita* polyp Baltic Sea husbandry	Marine Bouillon	30 °C	enrichment/ first screening
24	*Maribacter* sp.	MK967024.1	Bacteroidota	Flavobacteriia	Flavobacteriales	Flavobacteriaceae	*A. aurita* medusa Baltic Sea husbandry	Marine Bouillon	30 °C	enrichment/ first screening
57	*Olleya marilimosa*	MK967032.1	Bacteroidota	Flavobacteriia	Flavobacteriales	Flavobacteriaceae	*M. leidyi* Baltic Sea	Marine Bouillon	30 °C	enrichment/ first screening
79	*Olleya* sp.	MK967053.1	Bacteroidota	Flavobacteriia	Flavobacteriales	Flavobacteriaceae	*A. aurita* polyp Baltic Sea husbandry	Marine Bouillon	30 °C	enrichment/ first screening
181	*Chryseobacterium* sp.	MK967154.1	Bacteroidota	Flavobacteriia	Flavobacteriales	Weeksellaceae	Artificial Seawater 30 PSU	Marine Bouillon	30 °C	enrichment/ first screening
257	*Chryseobacterium* sp.	MK967218.1	Bacteroidota	Flavobacteriia	Flavobacteriales	Weeksellaceae	*M. leidyi* Baltic Sea husbandry	Marine Bouillon	30 °C	enrichment/ first screening
22	*Pseudolateromonas* sp.	MK967022.1	Pseudomonadota	Gammaproteobacteria	Alteromonadales	Pseudoalteromonadaceae	*A. aurita* medusa Baltic Sea husbandry	Marine Bouillon	30 °C	enrichment/ first screening
91	*Pseudoalteromonas prydzensis*	MK967065.1	Pseudomonadota	Gammaproteobacteria	Alteromonadales	Pseudoalteromonadaceae	*A. aurita* polyp Baltic Sea husbandry	Marine Bouillon	30 °C	enrichment/ first screening
101	*Pseudoalteromonas issachenkonii*	MK967075.1	Pseudomonadota	Gammaproteobacteria	Alteromonadales	Pseudoalteromonadaceae	*A. aurita* polyp North Sea husbandry	Marine Bouillon	30 °C	enrichment/ first screening
167	*Pseudoalteromonas* sp.	MK967140.1	Pseudomonadota	Gammaproteobacteria	Alteromonadales	Pseudoalteromonadaceae	Artificial Seawater 18 PSU	Marine Bouillon	30 °C	enrichment/ first screening
203	*Pseudoalteromonas espejiana*	MK967174.1	Pseudomonadota	Gammaproteobacteria	Alteromonadales	Pseudoalteromonadaceae	Artificial Seawater 30 PSU	Marine Bouillon	30 °C	enrichment/ first screening
219	*Pseudoalteromonas tunicata*	MK967188.1	Pseudomonadota	Gammaproteobacteria	Alteromonadales	Pseudoalteromonadaceae	*M. leidyi* Baltic Sea	Marine Bouillon	30 °C	enrichment/ first screening
224	*Pseudoalteromonas lipolytica*	MK967191.1	Pseudomonadota	Gammaproteobacteria	Alteromonadales	Pseudoalteromonadaceae	*M. leidyi* Baltic Sea	Marine Bouillon	30 °C	enrichment/ first screening
105	*Shewanella basaltis*	MK967079.1	Pseudomonadota	Gammaproteobacteria	Alteromonadales	Shewanellaceae	*A. aurita* polyp North Sea husbandry	Marine Bouillon	30 °C	enrichment/ first screening
21	*Cobetia amphilecti*	MK967021.1	Pseudomonadota	Gammaproteobacteria	Oceanospirillales	Halomonadaceae	*A. aurita* medusa Baltic Sea husbandry	Marine Bouillon	30 °C	enrichment/ first screening
55	*Marinomonas hwangdonensis*	MK967030.1	Pseudomonadota	Gammaproteobacteria	Oceanospirillales	Oceanospirillaceae	*M. leidyi* Baltic Sea	Marine Bouillon	30 °C	enrichment/ first screening
222	*Marinomonas pontica*	MK967189.1	Pseudomonadota	Gammaproteobacteria	Oceanospirillales	Oceanospirillaceae	*M. leidyi* Baltic Sea	Marine Bouillon	30 °C	enrichment/ first screening
262	*Oceanospirillaceae bacterium*	MK967222.1	Pseudomonadota	Gammaproteobacteria	Oceanospirillales	Oceanospirillaceae	*M. leidyi* Baltic Sea	Marine Bouillon	30 °C	enrichment/ first screening
11	*Pseudomonas* sp.	MK967012.1	Pseudomonadota	Gammaproteobacteria	Pseudomonadales	Pseudomonadaceae	*A. aurita* medusa Baltic Sea	Marine Bouillon	30 °C	enrichment/ first screening
90	*Pseudomonas putida*	MK967064.1	Pseudomonadota	Gammaproteobacteria	Pseudomonadales	Pseudomonadaceae	*A. aurita* polyp Baltic Sea husbandry	Marine Bouillon	30 °C	enrichment/ first screening
92	*Pseudomonas putida*	MK967066.1	Pseudomonadota	Gammaproteobacteria	Pseudomonadales	Pseudomonadaceae	*A. aurita* polyp Baltic Sea husbandry	Marine Bouillon	30 °C	enrichment/ first screening
93	*Pseudomonas* sp.	MK967067.1	Pseudomonadota	Gammaproteobacteria	Pseudomonadales	Pseudomonadaceae	*A. aurita* polyp Baltic Sea husbandry	Marine Bouillon	30 °C	enrichment/ first screening
94	*Pseudomonas* sp.	MK967068.1	Pseudomonadota	Gammaproteobacteria	Pseudomonadales	Pseudomonadaceae	*A. aurita* polyp Baltic Sea husbandry	Marine Bouillon	30 °C	enrichment/ first screening
132	*Pseudomonas fluorescens*	MK967106.1	Pseudomonadota	Gammaproteobacteria	Pseudomonadales	Pseudomonadaceae	Artificial Seawater 18 PSU	Marine Bouillon	30 °C	enrichment/ first screening
196	*Pseudomonas syringae*	MK967168.1	Pseudomonadota	Gammaproteobacteria	Pseudomonadales	Pseudomonadaceae	Artificial Seawater 30 PSU	Marine Bouillon	30 °C	enrichment/ first screening
77	*Vibrio anguillarum*	MK967051.1	Pseudomonadota	Gammaproteobacteria	Vibrionales	Vibrionaceae	*A. aurita* polyp Baltic Sea husbandry	Marine Bouillon	30 °C	enrichment/ first screening
80	*Vibrio anguillarum*	MK967054.1	Pseudomonadota	Gammaproteobacteria	Vibrionales	Vibrionaceae	*A. aurita* polyp Baltic Sea husbandry	Marine Bouillon	30 °C	enrichment/ first screening
18	*Staphylococcus aureus*	OQ398157	Bacillota	Bacilli	Bacillales	Staphylococcaceae	*A. aurita* medusa Baltic Sea husbandry	Marine Bouillon	30 °C	enrichment/ first screening
134	*Staphylococcus aureus*	OQ398164	Bacillota	Bacilli	Bacillales	Staphylococcaceae	Artificial Seawater 18 PSU	Marine Bouillon	30 °C	enrichment/ first screening
87	*Staphylococcus aureus*	OQ398160	Bacillota	Bacilli	Bacillales	Staphylococcaceae	*A. aurita* polyp Baltic Sea husbandry	Marine Bouillon	30 °C	enrichment/ first screening
14	*Staphylococcus warneri*	OQ398156	Bacillota	Bacilli	Bacillales	Staphylococcaceae	*A. aurita* medusa Baltic Sea	Marine Bouillon	30 °C	enrichment/ first screening
6	*Citrobacter freundii*	OQ398153	Pseudomonadota	Gammaproteobacteria	Enterobacterales	Enterobacteriaceae	*A. aurita* medusa Baltic Sea	Marine Bouillon	30 °C	enrichment/ first screening/ host range
7	*Citrobacter* sp.	OQ398154	Pseudomonadota	Gammaproteobacteria	Enterobacterales	Enterobacteriaceae	*A. aurita* medusa Baltic Sea	Marine Bouillon	30 °C	enrichment/ first screening/ host range
8	*Staphylococcus aureus*	PQ151711	Bacillota	Bacilli	Bacillales	Staphylococcaceae	*A. aurita* medusa Baltic Sea	Marine Bouillon	30 °C	enrichment/ first screening/ host range
62	*Sulfitobacter pontiacus*	OQ398158	Pseudomonadota	Alphaproteobacteria	Rhodobacterales	Rhodobacteraceae	*M. leidyi* Baltic Sea	Marine Bouillon	30 °C	host range
97	*Shewanella* sp.	OQ398161	Pseudomonadota	Gammaproteobacteria	Alteromonadales	Shewanellaceae	*A. aurita* polyp North Sea husbandry	Marine Bouillon	30 °C	host range
199	*Staphylococcus aureus*	OQ398168	Bacillota	Bacilli	Bacillales	Staphylococcaceae	Artificial Seawater 30 PSU	Marine Bouillon	30 °C	host range
DSMZ 11823	*Staphylococcus aureus*	DSMZ 11823	Bacillota	Bacilli	Bacillales	Staphylococcaceae	clinical material	Trypticase Soy Yeast Broth	37 °C	host range
67	*Staphylococcus aureus*	OQ398159	Bacillota	Bacilli	Bacillales	Staphylococcaceae	*M. leidyi* Baltic Sea husbandry	Marine Bouillon	30 °C	host range
102	*Staphylococcus aureus*	OQ398162	Bacillota	Bacilli	Bacillales	Staphylococcaceae	*A. aurita* polyp North Sea husbandry	Marine Bouillon	30 °C	host range
158	*Staphylococcus aureus*	OQ398165	Bacillota	Bacilli	Bacillales	Staphylococcaceae	Artificial Seawater 18 PSU	Marine Bouillon	30 °C	host range
161	*Staphylococcus aureus*	OQ398166	Bacillota	Bacilli	Bacillales	Staphylococcaceae	Artificial Seawater 18 PSU	Marine Bouillon	30 °C	host range
127	*Staphylococcus aureus*	OQ398163	Bacillota	Bacilli	Bacillales	Staphylococcaceae	*A. aurita* polyp North Atlantic husbandry	Marine Bouillon	30 °C	host range
DSMZ 28319	*Staphylococcus epidermidis*	DSMZ 28319	Bacillota	Bacilli	Bacillales	Staphylococcaceae	catheter sepsis	Trypticase Soy Yeast Broth	37 °C	host range
DSMZ 20328	*Staphylococcus hominis*	DSMZ 20328	Bacillota	Bacilli	Bacillales	Staphylococcaceae	human skin	Trypticase Soy Yeast Broth	37 °C	host range
DSMZ 100616	*Staphylococcus warneri*	DSMZ 100616	Bacillota	Bacilli	Bacillales	Staphylococcaceae	cleanroom facility, TAS	Trypticase Soy Yeast Broth	30 °C	host range
DSMZ 12643	*Streptococcus mitis*	DSMZ 12643	Bacillota	Bacilli	Lactobacillales	Streptococcaceae	oral cavity, human	Trypticase Soy Yeast Broth	37 °C	host range
DSMZ 20523	*Streptococcus mutans*	DSMZ 20523	Bacillota	Bacilli	Lactobacillales	Streptococcaceae	carious dentine	Trypticase Soy Yeast Broth	37 °C	host range
296	*Citrobacter braakii*	OQ398170	Pseudomonadota	Gammaproteobacteria	Enterobacterales	Enterobacteriaceae	*A. aurita* polyp North Atlantic husbandry	Marine Bouillon	30 °C	host range
283	*Citrobacter freundii*	OQ398169	Pseudomonadota	Gammaproteobacteria	Enterobacterales	Enterobacteriaceae	*A. aurita* polyp Baltic Sea husbandry	Marine Bouillon	30 °C	host range
321	*Citrobacter freundii*	OQ398172	Pseudomonadota	Gammaproteobacteria	Enterobacterales	Enterobacteriaceae	Artifical Seawater 30 PSU	Marine Bouillon	30 °C	host range
313	*Citrobacter* sp.	OQ398171	Pseudomonadota	Gammaproteobacteria	Enterobacterales	Enterobacteriaceae	Artifical Seawater 18 PSU	Marine Bouillon	30 °C	host range
DSMZ 18039	*Escherichia coli*	DSMZ 18039	Pseudomonadota	Gammaproteobacteria	Enterobacterales	Enterobacteriaceae	unknown source	Luria-Bertani Bouillon	37 °C	host range
strain 8	*Escherichia coli*	[47]	Pseudomonadota	Gammaproteobacteria	Enterobacterales	Enterobacteriaceae	unknown source	Luria-Bertani Bouillon	37 °C	host range
DSMZ 30083	*Escherichia coli*	DSMZ 30083	Pseudomonadota	Gammaproteobacteria	Enterobacterales	Enterobacteriaceae	urine	Luria-Bertani Bouillon	37 °C	host range
DSMZ 13698	*Escherichia fergusonii*	DSMZ 13698	Pseudomonadota	Gammaproteobacteria	Enterobacterales	Enterobacteriaceae	faeces of 1-year-old boy	Luria-Bertani Bouillon	37 °C	host range
strain 27	*Klebsiella oxytoca*	Prof. Dr. Podschun, (National Reference Laboratory for Klebsiella species, Kiel University)	Pseudomonadota	Gammaproteobacteria	Enterobacterales	Enterobacteriaceae	unknown source	Nutrient Broth	30 °C	host range
DSMZ 30104	*Klebsiella pneumoniae*	DSMZ 30104	Pseudomonadota	Gammaproteobacteria	Enterobacterales	Enterobacteriaceae	unknown source	Nutrient Broth	30 °C	host range
DSMZ 4782	*Shigella flexeneri*	DSMZ 4782	Pseudomonadota	Gammaproteobacteria	Enterobacterales	Enterobacteriaceae	unknown source	Caso Bouillon	37 °C	host range
DSMZ 1707	*Pseudomonas aeruginosa*	DSMZ 1707	Pseudomonadota	Gammaproteobacteria	Pseudomonadales	Pseudomonadaceae	unknown source	Caso Bouillon	30 °C	host range
9	*Staphylococcus aureus*	OQ398155	Bacillota	Bacilli	Bacillales	Staphylococcaceae	*A. aurita* medusa Baltic Sea	Marine Bouillon	30 °C	host range
170	*Staphylococcus aureus*	OQ398167	Bacillota	Bacilli	Bacillales	Staphylococcaceae	Artificial Seawater 18 PSU	Marine Bouillon	30 °C	host range
DSMZ 50256	*Pseudomonas syringae*	DSMZ 50256	Pseudomonadota	Gammaproteobacteria	Pseudomonadales	Pseudomonadaceae	*Triticum aestivum*, glume rot of wheat	Caso Bouillon	30 °C	host range
24	*Maribacter* sp.	MK967024.1	Bacteroidota	Flavobacteriia	Flavobacteriales	Flavobacteriaceae	*A. aurita* medusa Baltic Sea husbandry	Marine Bouillon	30 °C	host range
79	*Olleya* sp.	MK967053.1	Bacteroidota	Flavobacteriia	Flavobacteriales	Flavobacteriaceae	*A. aurita* polyp Baltic Sea husbandry	Marine Bouillon	30 °C	host range
98	*Olleya marilimosa*	MK967072.1	Bacteroidota	Flavobacteriia	Flavobacteriales	Flavobacteriaceae	*A. aurita* polyp North Sea husbandry	Marine Bouillon	30 °C	host range
108	*Chryseobacterium hominis*	MK967082.1	Bacteroidota	Flavobacteriia	Flavobacteriales	Flavobacteriaceae	*A. aurita* polyp North Sea husbandry	Marine Bouillon	30 °C	host range
57	*Olleya marilimosa*	MK967032.1	Bacteroidota	Flavobacteriia	Flavobacteriales	Flavobacteriaceae	*M. leidyi* Baltic Sea	Marine Bouillon	30 °C	host range
199	*Maribacter* sp.	MK967170.1	Bacteroidota	Flavobacteriia	Flavobacteriales	Flavobacteriaceae	Artificial Seawater 30 PSU	Marine Bouillon	30 °C	host range

**Table 2 viruses-16-01880-t002:** Viral genome characteristics and overview of assembly-related metrics.

Phage	NCBI Accession No.	No. of Reads	No. of Filtered Reads	Sequence Coverage	N50	Genome Length (bps)	GC Content (%)	Predicted ORFs	Unknown Proteins
***Staphylococcus* phage BSwM KMM1**	OP902294	4.085	3.214	247.488	17.553	137.386	31.77	259	200
***Citrobacter* phage BSwM KMM2**	OP902295	810	595	74.163	22.118	88.537	39.55	137	94
***Citrobacter* phage BSwS KMM3**	OP902292	837	598	130.676	20.517	49.164	43.17	92	58
***Citrobacter* phage BSwM KMM4**	OP902293	6.433	5.371	544.327	23.894	86.911	39.02	138	100
