# Supplementary material for: Four Novel Caudoviricetes Bacteriophages Isolated from Baltic Sea Water Infect Colonizers of Aurelia aurita
Source: Viruses. 2023 Jul 9;15(7):1525. doi: 10.3390/v15071525 (PMC10383413; doi:10.3390/v15071525)
Supplement: Supplementary file 1 [file viruses-15-01525-s001.zip › Supplementary Materials_caption.pdf]

Supplementary Materials

Figure S1. Transmission electron microscopy micrographs of isolated phages KMM1 - KMM4. (**A**) BSwM KMM1, (**B**) BSwM KMM2, (**C**) BSwS KMM3, (**D**) BSwM KMM4. (**i**) Overview image of undiluted phage lysates, scale bars represent 100 nm. (**ii-iv**) Intact virions. Orange arrows indicate a contractile tail and a blue arrow points to a non-contractile tail. Green triangles refer to icosahedral head shapes containing linear double-stranded DNA. Scale bar represents 50 nm.

Figure S2. Intergenomic similarities of phages KMM1 – KMM4. Heatmap generated after comparative genome analysis of isolated phages with their close homologs in BLASTn using VIRIDIC software. Intergenomic similarity values (upper triangle) and alignment indications are included in the heatmap (lower triangle and top bars). Three separate clusters, including KMM1 (OP902294) in cluster 1, KMM2 (OP902295) and KMM4 (OP902293) in cluster 2, and KMM3 (OP902292) in cluster 3, were formed based on an intergenomic similarity higher than the genus threshold of 70 % but lower than the species threshold of 95 %. Details on phage homologs and heatmap values are listed in **Table S1**.

Table S1. Details on phage homologs used for phylogenetic classification of KMM1-KMM4. (**Table sheet A**) Nearest homologs to isolated phages **KMM1 – KMM4** were identified with Vcontact2 and used for phylogenetic classification. (**Table sheet B**) Heatmap values were calculated using VIRIDIC software for intergenomic comparison and presented in **Figure S2**.

Table S2. Annotation of phage genomes KMM1 – KMM4. Annotations were derived from the EggNOG mapper and DRAM-V. Protein classifications and domain predictions were provided by InterPro.
