# Supplementary figures and images for: Four Novel Caudoviricetes Bacteriophages Isolated from Baltic Sea Water Infect Colonizers of Aurelia aurita
Source: Viruses. 2023 Jul 9;15(7):1525. doi: 10.3390/v15071525 (PMC10383413; doi:10.3390/v15071525)

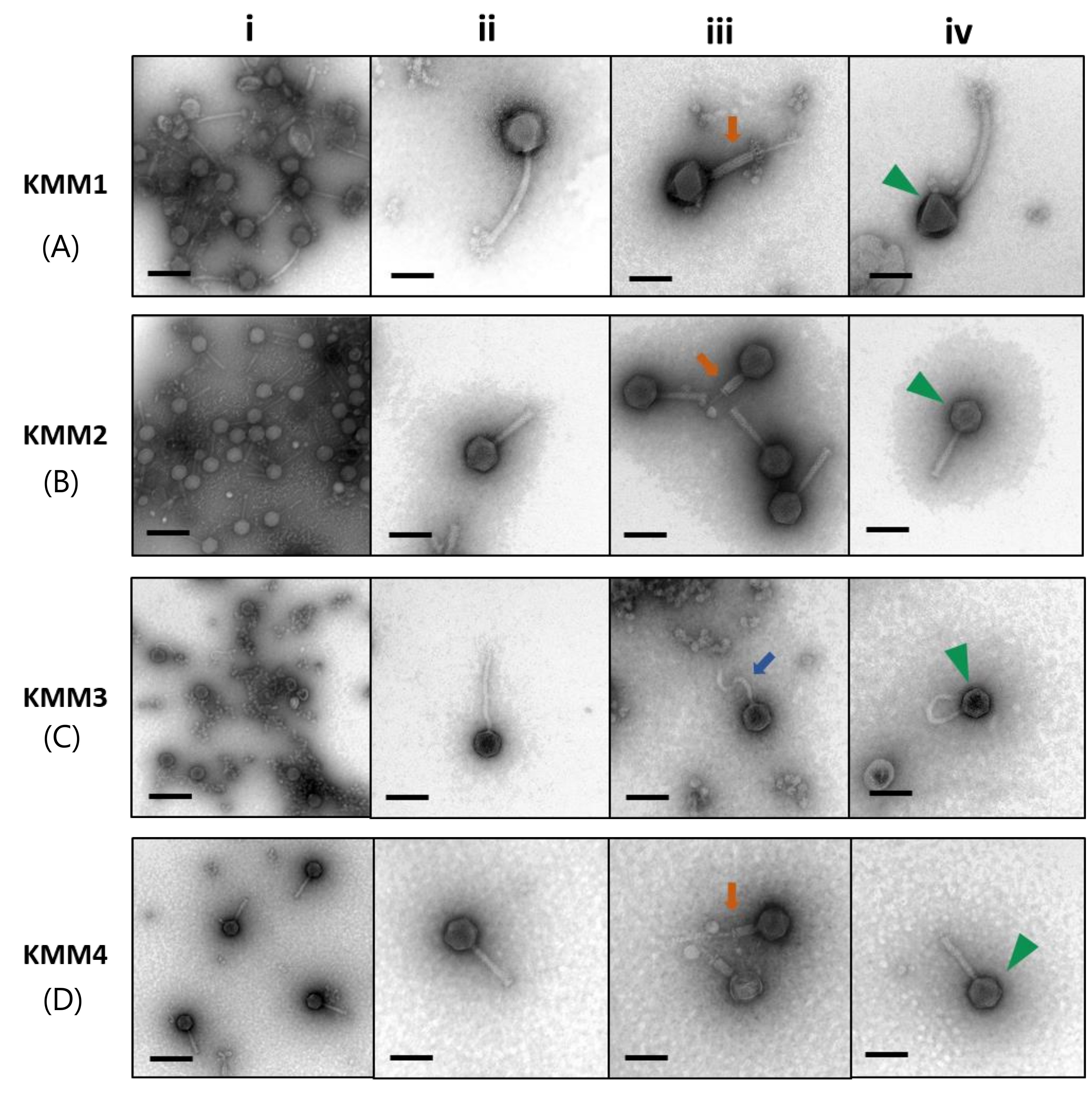

Supplement: Supplementary file 1 [file viruses-15-01525-s001.zip › Figure S1.jpg]
